# Immobilized Crosslinked Pectinase Preparation on Porous ZSM-5 Zeolites as Reusable Biocatalysts for Ultra-Efficient Hydrolysis of β-Glycosidic Bonds

**DOI:** 10.3389/fchem.2021.677868

**Published:** 2021-08-11

**Authors:** Can Liu, Liming Zhang, Li Tan, Yueping Liu, Weiqian Tian, Lanqing Ma

**Affiliations:** ^1^ Key Laboratory for Northern Urban Agriculture of Ministry of Agriculture and Rural Affairs, Beijing University of Agriculture, Beijing, China; ^2^ Department of Fibre and Polymer Technology, KTH Royal Institute of Technology, Stockholm, Sweden

**Keywords:** immobilization, pectinase, β-glycosidic bond, ZSM-5 zeolite, heat resistance, ethanol tolerance

## Abstract

In this study, we immobilized pectinase preparation on porous zeolite ZSM-5 as an enzyme carrier. We realized this immobilized enzyme catalyst, pectinase preparation@ZSM-5, *via* a simple combined strategy involving the van der Waals adsorption of pectinase preparation followed by crosslinking of the adsorbed pectinase preparation with glutaraldehyde over ZSM-5. Conformal pectinase preparation coverage of various ZSM-5 supports was achieved for the as-prepared pectinase preparation@ZSM-5. The porous pectinase preparation@ZSM-5 catalyst exhibited ultra-efficient biocatalytic activity for hydrolyzing the β-glycosidic bonds in the model substrate 4-nitrophenyl β-D-glucopyranoside, with a broad operating temperature range, high thermal stability, and excellent reusability. The relative activity of pectinase preparation@ZSM-5 at a high temperature (70 °C) was nine times higher than that of free pectinase preparation. Using thermal inactivation kinetic analysis based on the Arrhenius law, pectinase preparation@ZSM-5 showed higher activation energy for denaturation (315 kJ mol^−1^) and a longer half-life (62 min^−1^) than free pectinase preparation. Moreover, a Michaelis–Menten enzyme kinetic analysis indicated a higher maximal reaction velocity for pectinase preparation@ZSM-5 (0.22 µmol mg^−1^ min^−1^). This enhanced reactivity was attributed to the microstructure of the immobilized pectinase preparation@ZSM-5, which offered a heterogeneous reaction system that decreased the substrate–pectinase preparation binding affinity and modulated the kinetic characteristics of the enzyme. Additionally, pectinase preparation@ZSM-5 showed the best ethanol tolerance among all the reported pectinase preparation-immobilized catalysts, and an activity 247% higher than that of free pectinase preparation at a 10% (v/v) ethanol concentration was measured. Furthermore, pectinase preparation@ZSM-5 exhibited potential for practical engineering applications, promoting the hydrolysis of β-glycosidic bonds in baicalin to convert it into baicalein. This was achieved with a 98% conversion rate, i.e., 320% higher than that of the free enzyme.

## Introduction

The β-glycosidic bonds that link aglycone and glycosyl groups exist widely in secondary plant metabolites such as flavonoids (Xiao et al., 2014), triterpenes (Thimmappa et al., 2014), polyphenols (Marsol-Vall et al., 2016), and alkaloids (Sakamoto et al., 2020). The hydrolysis of β-glycosidic bonds is important for obtaining aglycone molecules, which have high bioactivity in a wide range of applications. However, conventional techniques for hydrolyzing β-glycosidic bonds are conducted under strongly acidic and high-temperature conditions, which have detrimental effects on the bioactivity of the as-prepared glycosides and the environment (Hoang et al., 2018). In contrast, enzyme hydrolysis implemented under mild conditions is considered a promising alternative technique to replace traditional hydrolysis techniques for glycosidic bonds in industrial engineering (Cao et al., 2015). For example, pectinase preparation is used to hydrolyze α-glycosidic bonds in pectin during industrial production of juices (Sprockett et al., 2011), which can efficiently improve the yield and purity of the juices while reducing their viscosity (John et al., 2020; Tavares et al., 2020). Flavonoids are an important class of secondary metabolites in plants with anti-cancer (Zhang et al., 2003; Cheng et al., 2018), anti-inflammatory (Yoon et al., 2009), lipid-regulating (Seo et al., 2014), and antioxidant (Wang et al., 2014) functions. In plants, flavonoids mostly exist in the form of flavonoid glycosides rather than flavonoid aglycones (Offen et al., 2006; Le Roy et al., 2016). However, flavonoid aglycones usually have higher biological activity than flavonoid glycosides. Therefore, the hydrolysis of flavonoid glucosides to produce high-value flavonoid aglycones is an important engineering application. Pectinase preparation can also hydrolyze the β-glycosidic bonds of flavonoid glycosides and transform flavonoid glycosides into the corresponding aglycones while avoiding the oxidation of flavonoid molecules (Vallance et al., 2019; Cao et al., 2020).

However, the industrial applications of enzymatic hydrolysis are limited by the reusability of free enzymes, which is typically poor because of the ease of enzyme deactivation and the difficulty of separating the enzyme from final products. Notably, the immobilization of enzymes on solid supports offers a promising approach to enhance the reusability and stability of enzymes (Mateo et al., 2007; Skoronski et al., 2014), thereby reducing costs and promoting the development of enzyme hydrolysis techniques for industrial applications. For instance, Zeolite Socony Mobil–5 (ZSM-5) (Mitchell and Pérez-Ramírez, 2011), a porous zeolite with a large specific surface area, is nontoxic and inexpensive, and it is used as a support to immobilize enzymes (Kumari et al., 2015). Such zeolites not only act as skeletons but also have the potential to enhance the catalytic performance of immobilized enzymes by shifting the optimal temperature, stability, and pH values (Min and Yoo, 2014; Liu et al., 2017).

Previous studies on immobilized enzymes have mainly considered aqueous reaction environments; for example, pectinase preparation has been immobilized over polyvinyl alcohol (PVA) gels, chitosan particles, and docusate sodium salt (AOT)-Fe_3_O_4_ for use in juice production (Wang et al., 2013; Cerreti et al., 2017; John et al., 2020). However, the heterogeneity of the immobilized pectinase preparation catalysts and organic substrates within aqueous mediums limits the application scope, particularly for nonpolar substrates. Interestingly, organic solvents such as ethanol can facilitate the dissolution of nonpolar substrates in aqueous solutions, which can promote mass transfer between pectinase preparation and the substrate (Qu et al., 2016). However, the ethanol tolerance of immobilized pectinase preparation has not been investigated so far.

**FIGURE 1:**
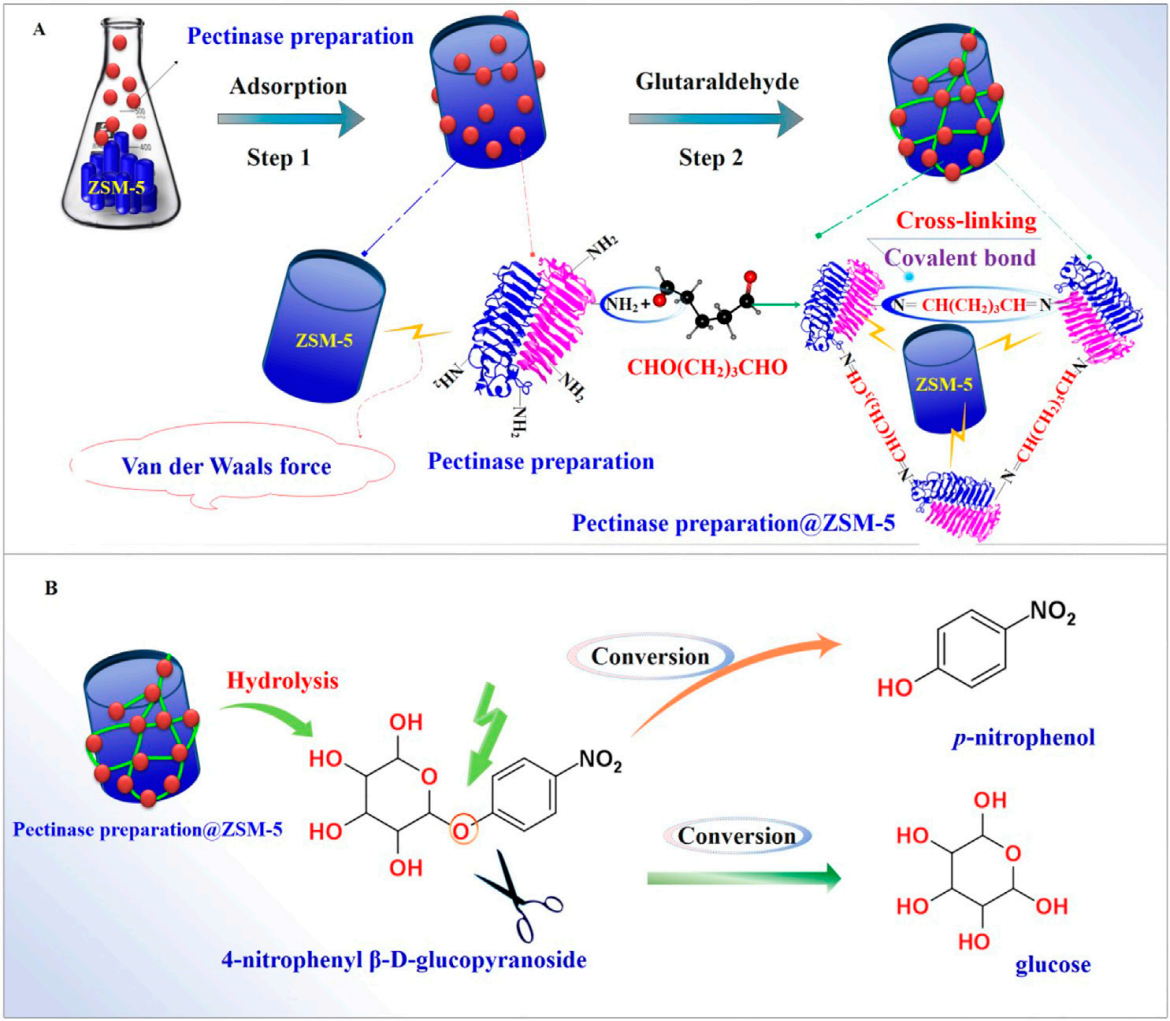
Fabrication and application of the pectinase preparation@ZSM-5 catalysts. Schematic diagrams of **(A)** pectinase preparation@ZSM-5 fabrication and **(B)** catalytic hydrolysis of the model substrate 4-nitrophenyl β-D-glucopyranoside (PNPG) using pectinase preparation@ZSM-5.

Therefore, in this study, we fabricated the enzyme catalyst pectinase preparation@ZSM-5 by immobilizing pectinase preparation over porous ZSM-5 zeolites that acted as enzyme carriers. We created a simple combined strategy in which the pectinase preparation was first adsorbed *via* van der Waals forces onto ZSM-5 before crosslinking between the adsorbed pectinase preparation and glutaraldehyde occurred (Figure 1A). We evaluated the biocatalytic capability of the as-prepared pectinase preparation@ZSM-5 to hydrolyze β-glycosidic bonds using a model reaction, namely, the hydrolysis of 4-nitrophenyl β-D-glucopyranoside (PNPG) into *p*-nitrophenol and sugar ligands (Figure 1B). Furthermore, we demonstrated the practical applicability of the pectinase preparation@ZSM-5 biocatalysts for hydrolyzing β-glycosidic bonds by converting flavonoid glycoside (baicalin) into flavonoid aglycone (baicalein), which was shown to possess higher antioxidant activity than baicalin.

## Experimental Section

### Chemicals

Three ZSM-5 zeolites, ZSM-5(27), ZSM-5(85), and ZSM-5(500), with Si:Al molar ratios of 27, 85, and 500, respectively, were purchased from Nankai University Catalyst Co., Ltd. Pectinase preparation (20 U/mg d.w.) was obtained from Sangon Biotech Co., Ltd. (Shanghai). The chemicals 4-nitrophenyl β-D-glucopyranoside (PNPG) and catalase (CAT) and kits for SOD activity detection and glutathione (GSH) content detection were purchased from Beijing Solarbio Science & Technology Co., Ltd. Baicalin (HPLC grade) was purchased from Aladdin Industrial Corporation. RAW264.7 cells were obtained from Peking Union Medical College. Dulbecco’s modified Eagle’s medium (DMEM) was purchased from Beijing Solarbio Science & Technology Co., Ltd. All other chemicals were of analytical grade.

### Fabrication of Pectinase Preparation@ZSM-5 Catalysts

We prepared the pectinase preparation@ZSM-5 catalysts as follows. First, 100 mg of pectinase preparation from *Aspergillus niger* was dissolved in 10 ml of acetate buffer solution (pH 5, 20 mM), and then 0.5 g of ZSM-5 zeolite was added. The mixed dispersion was continuously shaken for 12 h to ensure adequate adsorption of pectinase preparation onto the surface of the ZSM-5 support. The final pectinase preparation@ZSM-5 catalyst was obtained after crosslinking the pectinase preparation on the ZSM-5 surface by immersion in a glutaraldehyde solution (0.5%) for 4 h at 25°C, with stirring at 120 r min^−1^. The residual uncrosslinked pectinase preparation was removed by continuous washing until no protein was detected in the washing solution. To detect the protein concentration, the Bradford method was used with bovine serum albumin as the standard (Bradford, 1976). The amount of pectinase preparation immobilized on the zeolite was calculated from the difference between the initial amount of pectinase preparation and the amount of un-crosslinked pectinase preparation.

### Characterization of Pectinase Preparation@ZSM-5 Catalysts

The morphologies of the pectinase preparation@ZSM-5 catalysts were characterized by SEM (JSM-6700F, JEOL, Japan). X-ray powder diffraction (XRD; D8, Bruker, Germany) was conducted at 40 kV and 40 mA with Cu Kα radiation. N_2_ adsorption and desorption isotherms of the pectinase preparation@ZSM-5 catalysts at 77 K were measured using a nitrogen analyzer (Autosorb-iQ-MP, Quantachrome Co., United States). The specific surface areas and pore size distributions of the pectinase preparation@ZSM-5 catalysts were calculated *via* Brunauer–Emmett–Teller (BET) and density functional theory (DFT) analyses (Liu et al., 2013; Jayan and Islam, 2020). Fourier transform infrared (FTIR) spectra were acquired using a spectrophotometer in diffuse reflectance spectroscopy mode (IR Tracer-100, Shimadzu Co., Japan). The ammonia temperature-programmed desorption (NH_3_-TPD) curves of the pectinase preparation@ZSM-5 catalysts were acquired using a chemisorption analyzer (Auto Chem1 II 2920, Micromeritics Instrument Corp., United States) as follows: the catalyst (0.15 g) was pretreated at 400°C for 30 min under an N_2_ atmosphere and was then cooled to room temperature. The pretreated catalyst was equilibrated with NH_3_ gas for 1 h at 800°C with heating at a rate of 10°C min^−1^, and then, the excess physisorbed NH_3_ was eliminated by placing the catalyst under N_2_ flow for 30 min.

### Evaluation of Hydrolysis Activity Toward β-Glycosidic Bonds of Pectinase Preparation@ZSM-5 Catalysts

We used enzymatic hydrolysis of 4-nitrophenyl β-D-glucopyranoside (PNPG) as a model reaction to evaluate the biocatalytic activity of pectinase preparation@ZSM-5 catalysts for the hydrolysis of β-glycosidic bonds (Zhang et al., 2003; Zhang et al., 2018). In brief, 1 ml of 50 mmol L^−1^ PNPG in a pH 2–6 buffer solution was mixed with 50 mg of the pectinase preparation@ZSM-5 catalyst in a reaction tube, and the mixed solution was incubated at 30–70°C for 10–50 min. Subsequently, 50 μl of this hydrolysis solution was mixed with 100 μl of Na_2_CO_3_ solution (1 M) to terminate the enzymatic reaction. The obtained mixture was filtered to remove the catalysts, and thereafter, it was characterized using UV–visible spectrophotometry at 400 nm. One unit of enzyme activity was defined as the amount of enzyme that generates 1 μmol min^−1^ of *p*-nitrophenol under the conditions described. We analyzed the effect of ethanol on enzyme activity by adding ethanol to the substrate solution at final concentrations in the range of 0–25% (v/v) at a given time.

### Thermal Stability Analysis for the Enzyme Catalysts of Pectinase Preparation@ZSM-5

We conducted thermal inactivation kinetic analysis of the pectinase preparation@ZSM-5 catalysts under different high-temperature conditions. The decay constant, *k*_d_, is defined using the following equation:

$$\;\text{ln}\frac{V_{t}}{V_{0}}=-k_{\text{d}}\times t,$$ (1)

where *V*_*t*_ and *V*_0_ are the enzyme activity at the time points *t* and *t*_0_, respectively.

The half-life *t*_1/2_ for the thermal deactivation of an enzyme catalyst is related to *k*_d_ as follows:

$$t_{1/2}=\frac{\text{ln}2}{k_{\text{d}}}.$$ (2)

The decimal reduction time, D-value, is calculated as follows:

$$D=\frac{\text{ln}10}{k_{\text{d}}},$$ (3)

where *k*_d_ is the decay constant for the first-order kinetics at a given temperature.

The activation energy required to denature an enzyme, *E*_d_, is calculated from the Arrhenius law (Vishwasrao and Ananthanarayan, 2018), which describes the temperature dependence of the rate constant:

$$\text{ln}\frac{k_{\text{d}2}}{k_{\text{d}1}}=-\frac{E_{\text{d}}}{R}\left(\frac{1}{T_{2}}-\frac{1}{T_{1}}\right),$$ (4)

where *R* is the ideal gas constant [8.314 J·(Kmol)^−1^] and *k*_d1_ and *k*_d2_ are the decay rate constants for the thermal exposure of the enzyme at temperatures *T*_1_ and *T*_2_, respectively.

### Evaluation of Reusability of Pectinase Preparation@ZSM-5 Catalysts

We evaluated the reusability of the three pectinase preparation@ZSM-5 catalysts by testing their hydrolytic activity toward β-glycosidic bonds over 10 cycles of the reaction. In brief, pectinase preparation@ZSM-5 was incubated with the PNPG substrate at pH 3 and 42°C for 60 min. The pectinase preparation@ZSM-5 catalysts were then separated from the substrate solution after each hydrolytic reaction by centrifuging and washing three times with 3 ml of 20 mM acetate buffer solution (pH 3.0). The recycled pectinase preparation@ZSM-5 catalyst was resuspended in the reaction tube for the next cycle to be run under the same conditions. The relative activity of the recycled pectinase preparation@ZSM-5 catalyst was evaluated as a percentage of the retained activity, accounting for the initial activity.

### Kinetic Parameter Analysis of Pectinase Preparation@ZSM-5 Catalysts

We analyzed the kinetic parameters of pectinase preparation@ZSM-5 using the hydrolysis of PNPG as a model reaction. The substrate PNPG concentration was varied from 2 to 25 mM in acetate buffer solution (20 mM). The substrate was allowed to equilibrate for 10 min, and then 1 ml of the substrate solution was mixed with 50 mg of pectinase preparation@ZSM-5 catalyst in a reaction tube for 10 min at 50°C. The mixture was then filtered to remove the catalyst, and the filtrate was characterized by detecting its absorption intensity at 400 nm using a UV–visible spectrophotometer. The Michaelis–Menten constant, *K*_m_, and maximum reaction rate, *V*_max_, were calculated from the Michaelis–Menten and Hanes–Woolf plots.

The Michaelis–Menten kinetics was fitted using the following equation:

$$V=\frac{V_{\text{max}}.\text{S}}{\text{S}+K_{\text{m}}},$$ (5)

where *V* is the reaction rate and *S* is the concentration of the substrate (mM); *K*_m_ and *V*_max_ values were evaluated from the Hanes–Woolf double-reciprocal plots to fit the following equation:

$$\frac{1}{V}=\frac{K_{\text{m}}}{V_{\text{max}}}\cdot \frac{1}{\left[\text{S}\right]}+\frac{1}{V_{\text{max}}},$$ (6)

### Application of Enzyme to Biotransformation of Baicalin

Baicalin (5 µM) and the pectinase preparation catalyst were mixed in acetate buffer (20 mM, pH 5) with 10% ethanol, and the mixture was incubated for 24 h at 42°C with shaking at 120 rpm. Then, the pectinase preparation catalyst was separated by filtration. The conversion product in the filtrate was analyzed using a Prominence LC-20 A HPLC instrument with a Waters C18 column (4.6 × 150 mm) and photodiode array (PDA) detector at 265 nm (Shimadzu, Japan). The mobile phase was a mixture of aqueous phosphoric acid (0.2%) and methanol, and the gradient elution flow rate was 1.0 ml/min (0–10 min, 25–100% methanol; 10–14 min, 100% methanol; 14–18 min, 100–25% methanol). The molecular weight of the product was identified using an Agilent 1290LC-6470 A-QQQ-MS mass spectrometer with an AJS-ESI electrospray ionization source (Agilent Corporation, MA, United States). The mass spectrometer was operated in negative ion mode for detection with an ion source temperature of 300°C, gas flow of 5 L min^−1^, capillary voltage of 3500 V, sheath gas (N_2_) temperature of 250°C, and sheath gas flow rate of 11 L min^−1^. N_2_ was used as the collision gas and the collision energy was 35 eV for the baicalein product.

### Cell Biology Experiments

Mouse mononuclear macrophages (RAW264.7) were obtained from the School of Basic Sciences, Peking Union Medical College, Beijing, China. The cells were inoculated in DMEM with glucose (4.5 g L^−1^), penicillin (100 U ml^−1^), streptomycin (100 U ml^−1^), and 10% (v/v) fetal bovine serum and were cultured at 37°C with a CO_2_ saturation level of 5%. When the cells had reached the logarithmic growth phase, 200 μl of the medium was added to individual wells in a 96-well plate (2 × 10^4^ cells seeded per well). The cells were treated with lipopolysaccharide (LPS, 10 μg ml^−1^) as a positive control to establish an inflammatory cell model. The LPS-injured cells were treated with baicalein and baicalin at final drug concentrations of 1, 10, 50, 100, and 200 μg ml^−1^ for 24 h. The cells were then centrifuged at 1000 r min^−1^ for 5 min, collected, added to a lysis solution for lysis, and then centrifuged at 8,000 r min^−1^ for 10 min. After centrifugation at 4°C for an additional 10 min, the CAT, SOD, and GSH contents of the supernatant were determined using the aforementioned kits, according to the manufacturer’s instructions. Intracellular oxygen-free radicals in the cells were measured using a dichlorofluorescein (DCFH) fluorescence probe and confocal microscopy (Huo et al., 2017). Oxygen radicals in the cells were detected using fluorescent probes as follows. DMEM with DCFH-DA at a final concentration of 10 μmol L^−1^ was added to the cultured cells and then they were incubated for 1 h. The medium was extracted, and the cells were washed three times with DMEM to remove excess probe molecules. Light exposure was avoided throughout these steps. The cells were observed spectroscopically with excitation light at a wavelength of 488 nm, and the fluorescence at an emission wavelength of 525 nm was monitored using an inverted fluorescence microscope (Axio Observer A1, Carl Zeiss, Germany). The brighter the fluorescence in the cells, the higher their oxygen-free radical content (Zhang et al., 2019).

### Statistical Analysis

The results in this work were presented as means ± standard deviations (SD) calculated from values obtained from three independent experiment repetitions. Statistical analysis was performed using SPSS software, and statistical significance was estimated using analysis of variance (ANOVA).

## Results and Discussion

### Fabrication and Characterization of Pectinase Preparation@ZSM-5

**FIGURE 2:**
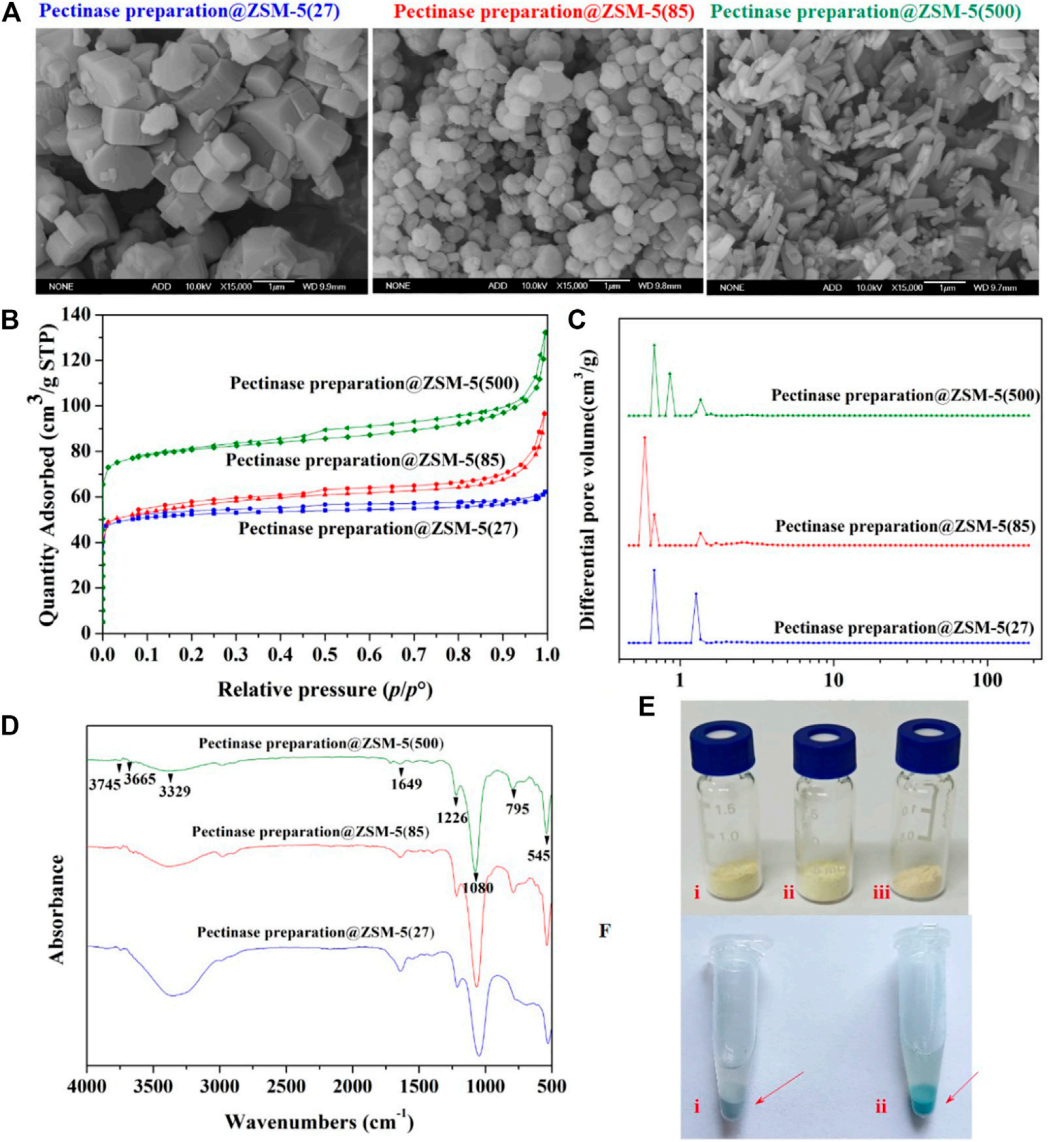
Microstructure characterization for the pectinase preparation@ZSM-5 catalysts. **(A)** SEM images of pectinase preparation@ZSM-5(27), pectinase preparation@ZSM-5(85), and pectinase preparation@ZSM-5(500). **(B)** Nitrogen adsorption and desorption isotherms, and **(C)** corresponding pore-size distributions of pectinase preparation@ZSM-5 catalysts. **(D)** FT-IR spectra of pectinase preparation@ZSM-5. **(E)** Photographs of (i) pectinase preparation@ZSM-5 (27), (ii) pectinase preparation@ZSM-5(85), and (iii) pectinase preparation@ZSM-5(500). **(F)** Photographs of (i) ZSM-5 and (ii) pectinase preparation@ZSM-5 stained with Coomassie brilliant blue.

We fabricated pectinase preparation@ZSM-5 using a combined two-step process (Figure 1A) in which the pectinase preparation was first physisorbed on the ZSM-5 carriers by van der Waals forces and was then crosslinked using glutaraldehyde to obtain immobilized pectinase preparation on the ZSM-5 surface (Figure 2E). Three ZSM-5 zeolites with different Si:Al molar ratios of 27, 85, and 500, referred to as ZSM-5(27), ZSM-5(85), and ZSM-5(500), were used to immobilize pectinase preparation, yielding three different products denoted as pectinase preparation@ZSM-5(27), pectinase preparation@ZSM-5(85), and pectinase preparation@ZSM-5(500), respectively.

We used the Coomassie brilliant blue dye to confirm the incorporation of pectinase preparation into the final catalyst products. Figure 2F shows the dark blue color of pectinase preparation@ZSM-5 after it was stained with Coomassie brilliant blue. The reaction between the Coomassie brilliant blue dye molecules and the enzyme proteins resulted in this color change. This result suggests that the pectinase preparation enzyme was successfully immobilized on the ZSM-5 surface. We used Fourier transform infrared spectroscopy (FTIR) to further characterize the immobilization of pectinase preparation on ZSM-5 (Figure 2D). All the pectinase preparation@ZSM-5 products showed similar characteristic peaks originating from the ZSM-5 support (Tabor et al., 2019; Yi et al., 2019), including peaks at 3,745 cm^−1^ assigned to the Si–OH bond, at 3,665 cm^−1^ to the Si–OH–Al moiety, at 3,329 cm^−1^ to the O–H bond, and at 1226 cm^−1^, 1080 cm^−1^, and 795 cm^−1^ to the T–O–T (T = zeolite tetrahedron) group. Notably, the FTIR spectra of all the pectinase preparation@ZSM-5 catalysts show a characteristic peak at 1649 cm^−1^, which is assigned to vibration of the C–N moiety in pectinase preparation (Lazarev et al., 1985; Ji et al., 2020). These results further indicated the successful immobilization of the enzyme on the supports.

Scanning electron microscopy (SEM) images (Figure 2A **)** of pectinase preparation@ZSM-5 showed that the pectinase preparation was uniformly deposited on the surface of the different ZSM-5 scaffolds without obvious aggregation of the enzyme particles. This conformal coverage offered the structural features required for efficient enzymatic catalysis. The ZSM-5 supports with different Si:Al molar ratios possessed different morphologies, as shown in Figure 2A. The structures of ZSM-5(27), ZSM-5(85), and ZSM-5(500) were blocky, spherical, and rod-shaped, respectively, offering different surface areas to anchor the pectinase preparation. The nitrogen adsorption and desorption isotherms of the pectinase preparation@ZSM-5 (Figure 2B) resembled typical type-IV curves (Piumetti et al., 2015) and indicated large specific surface areas of 207 m^2^ g^−1^ for pectinase preparation@ZSM-5(27), 213 m^2^ g^−1^ for pectinase preparation@ZSM-5(85), and 317 m^2^ g^−1^ for pectinase preparation@ZSM-5(500) (Supplementary Table 2). The corresponding pore size distributions (Figure 2C) of the pectinase preparation@ZSM-5 catalysts suggested the presence of hierarchical pore systems mainly consisting of micropores and small mesopores, which can promote mass transport during enzymatic catalysis.

### Enzymatic Hydrolysis Activity of Pectinase Preparation@ZSM-5

**FIGURE 3:**
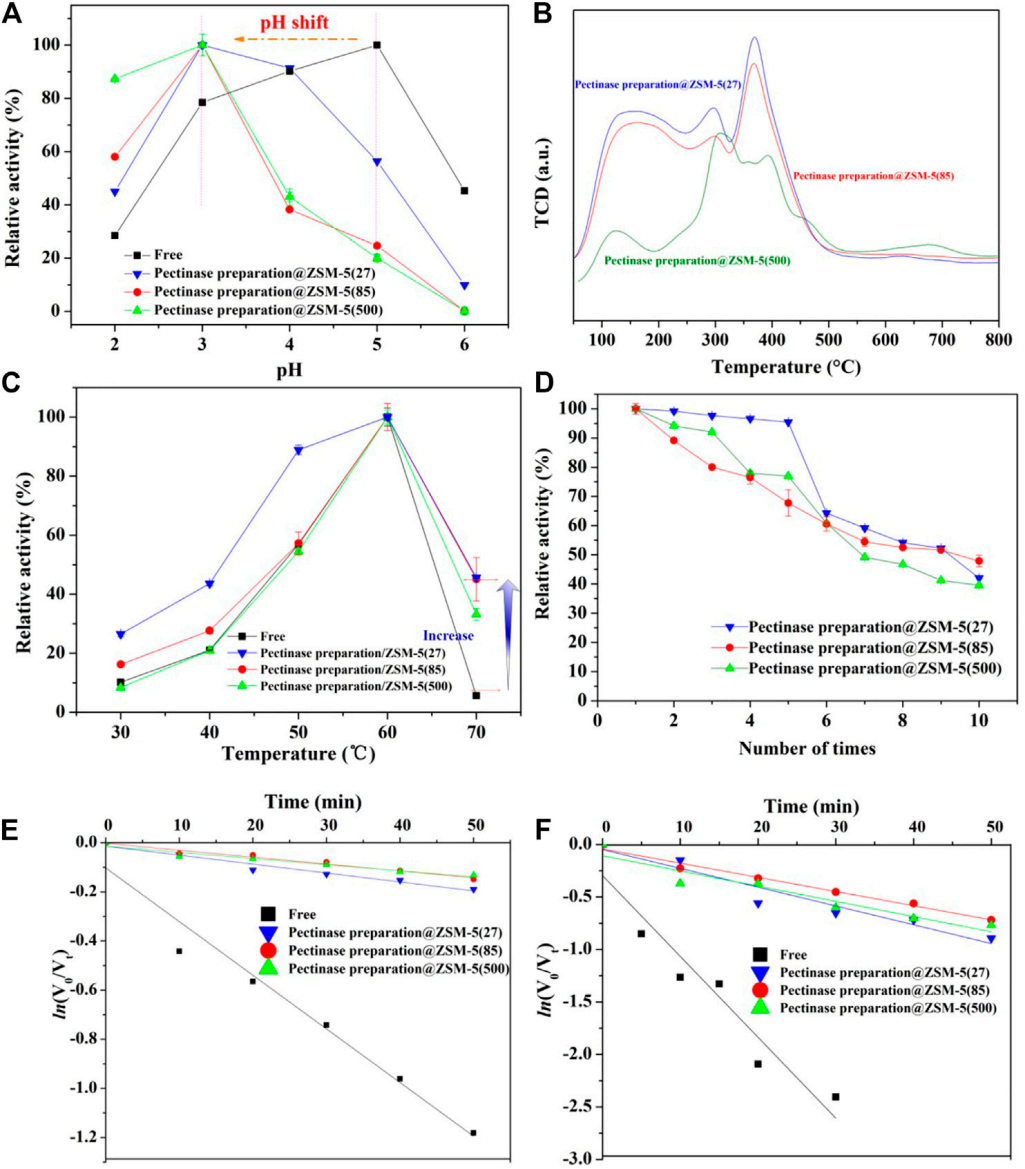
Evaluation of hydrolysis performance of pectinase preparation@ZSM-5. **(A)** Effect of pH of the reaction medium on the hydrolytic activity of pectinase preparation@ZSM-5 catalysts and free pectinase preparation. **(B)** NH_3_-TPD profiles of pectinase preparation@ZSM-5 catalysts. **(C)** Effect of reaction medium temperature on the hydrolytic activity of pectinase preparation@ZSM-5 catalysts and free pectinase preparation. **(D)** Reusability of pectinase preparation@ZSM-5 catalysts. **(E,F)** First-order thermal deactivation plots of pectinase preparation@ZSM-5 catalysts and free pectinase preparation at **(E)** 60°C and **(F)** 65°C.

We evaluated the enzymatic activity of pectinase preparation@ZSM-5 for the hydrolysis of β-glycosidic bonds using a model reaction, the conversion of 4-nitrophenyl β-D-glucopyranoside (PNPG) into *p*-nitrophenol and sugar ligands (Figure 1B). We first investigated the effect of the pH of the reaction medium on the biocatalytic activity of pectinase preparation@ZSM-5 (Figure 3A). The optimum pH for pectinase preparation@ZSM-5 was found to be 3, which is lower than that for the free enzyme (pH = 5). This shift in the optimal pH value toward a lower value indicates that more acidic conditions are favored after the immobilization of pectinase preparation. This effect was attributed to the change in the microenvironment of the enzyme activity center resulting from the interaction between the pectinase preparation and the ZSM-5 carrier. The ammonia temperature-programmed desorption (NH_3_-TPD) spectrum indicated a high acid content of 2.78 mmol g^−1^ for pectinase preparation@ZSM-5(85) (Figure 3B and Supplementary Table 2). Thus, the NH_3_-TPD results proved the existence of an acidic microenvironment in pectinase preparation@ZSM-5 (Haag et al., 1984; Kang et al., 2020; Bayramoglu et al., 2007). These findings are consistent with previously reported results on enzyme-immobilized catalysts (Carević et al., 2016).

We also evaluated the reaction temperature, which is another important parameter that affects enzyme activity. As shown in Figure 3C, all the pectinase preparation@ZSM-5 catalysts showed enhanced activities as the reaction temperature increased. The activities were maximized at 60°C, similar to the trend for free pectinase preparation because the increase in reaction temperature accelerated substrate movement and hence accelerated the reaction. Notably, the relative activity of the pectinase preparation@ZSM-5 series of catalysts was nine times higher than that of free pectinase preparation at a high temperature (70°C). In general, such high temperatures can denature and inactivate enzymes. These findings suggested that the ZSM-5 carrier effectively acted as a buffer that minimized the negative effects of high reaction temperature on enzyme activity. The immobilization of pectinase preparation on ZSM-5 resulted in a broader operating temperature range than that of free pectinase preparation.

We studied the thermal inactivation kinetics to understand the mechanism responsible for the high activity of pectinase preparation@ZSM-5 at high temperatures. Semi-logarithmic plots of the residual activity versus heating time exhibited a linearity (*R* ^2^ > 0.9) (Figures 3E,F), indicating that a first-order kinetic model could be used to fit the thermal deactivation kinetics of pectinase preparation. The kinetic deactivation parameters calculated using Eqs. 1–4 (see Experimental Section), including the decay constant (*k*_d_), half-life (*t*_1/2_), *D*-value, and activation energy for denaturation (*E*_*d*_), are listed in Table 1. *k*_*d*_ was used to reveal the inactivation velocity of the enzyme (Labus et al., 2015; Illeová et al., 2020), which was calculated from the slope of the plot of the natural logarithm of the residual enzyme activity versus thermal exposure time. The *k*_*d*_ values of pectinase preparation@ZSM-5 increased dramatically as the temperature increased from 333 to 338 K (Table 1), suggesting a faster inactivation velocity at a higher temperature. The *t*_1/2_ and D-value represent the time *t* required for the enzyme activity to decrease to half and one-tenth of its initial values at a given temperature, respectively. The *t*_1/2_ and D-value of each catalyst sample decreased significantly with temperature (Table 1). *E*_*d*_ is an index of the energy required for enzyme denaturation, calculated using the Arrhenius law. We observed lower *k*_*d*_ but larger *t*_1/2_, D-value, and *E*_*d*_ for all the pectinase preparation@ZSM-5 catalysts than the corresponding values for free pectinase preparation, indicating higher thermal stability for the pectinase preparation@ZSM-5 than for free pectinase preparation. More interestingly, pectinase preparation@ZSM-5(85) showed the highest *E*_*d*_ value of 315 kJ mol^−1^, suggesting that it has the best thermal stability among the as-prepared pectinase preparation@ZSM-5 catalysts (Rapeanu et al., 2005; Verhaeghe et al., 2016; Abdel Wahab et al., 2018; Ahmed et al., 2018). It has been reported in the literature (Opalka and Zhu, 2016; Fujitsuka et al., 2020) that high aluminum to silicon ratio in ZSM-5 can improve the adsorption effect of molecular sieve and enhance the van der Waals force between ZSM-5 and other molecules. The high aluminum contents of ZSM-5(85) and ZSM-5(500) indicate that the van der Waals force between the ZSM-5 carrier and enzyme is stronger than that of ZSM-5(27), and the enzyme is more tightly bound to the carrier. When subjected to heat treatment, the ZSM-5 carrier acts as a backbone, protecting the protein molecular structure from being easily destroyed.

**TABLE 1:** Kinetic parameters of pectinase preparation@ZSM-5 and free pectinase preparation.

Samples	Kinetic parameters of thermal deactivation							Kinetic parameters	
	*k*_d_ [min^−1^]		*t*_1/2_ [min^−1^]		D-value [min]		*E*_d_ (kJ)	*K*_m_ (µM)	*V*_max_ [µmol mg^−1^ min^−1^]
Temperature	333 K	338 K	333 K	338 K	333 K	338 K			
Free	0.0218	0.077	31.75	9.00	105.48	29.89	236	3.06	0.12
Pectinase preparation@ZSM-5(27)	0.0036	0.018	198.04	36.95	657.88	122.74	300	4.42	0.17
Pectinase preparation@ZSM-5(85)	0.0025	0.014	277.26	61.89	921.03	205.59	315	3.84	0.22
Pectinase preparation@ZSM-5(500)	0.0028	0.015	246.67	47.84	819.43	158.91	306	3.92	0.11

Note: ***k***_**d**_ is the decay constant, ***t***_**1/2**_ is the half-life, the **D-value** is the time required for the enzyme activity to decrease to one-tenth of its initial value, ***E***_**d**_ is the activation energy required to denature an enzyme, ***K***_**m**_ is the Michaelis–Menten constant, and ***V***_**max**_ is the maximum reaction rate.

We investigated the reusability of the three immobilized pectinase preparation@ZSM-5 catalysts *via* repeated hydrolysis of PNPG **(** Figure 3D **)**. We observed that the residual activity of each pectinase preparation@ZSM-5 continuously decreased with the cycle number, but approximately 40% of the initial activity was retained after ten cycles. The decrease in enzyme activity could be attributed to the inactivation and peeling off of the immobilized pectinase preparation from the ZSM-5 support during cycling.

### Kinetic Parameters of Pectinase Preparation@ZSM-5

**FIGURE 4:**
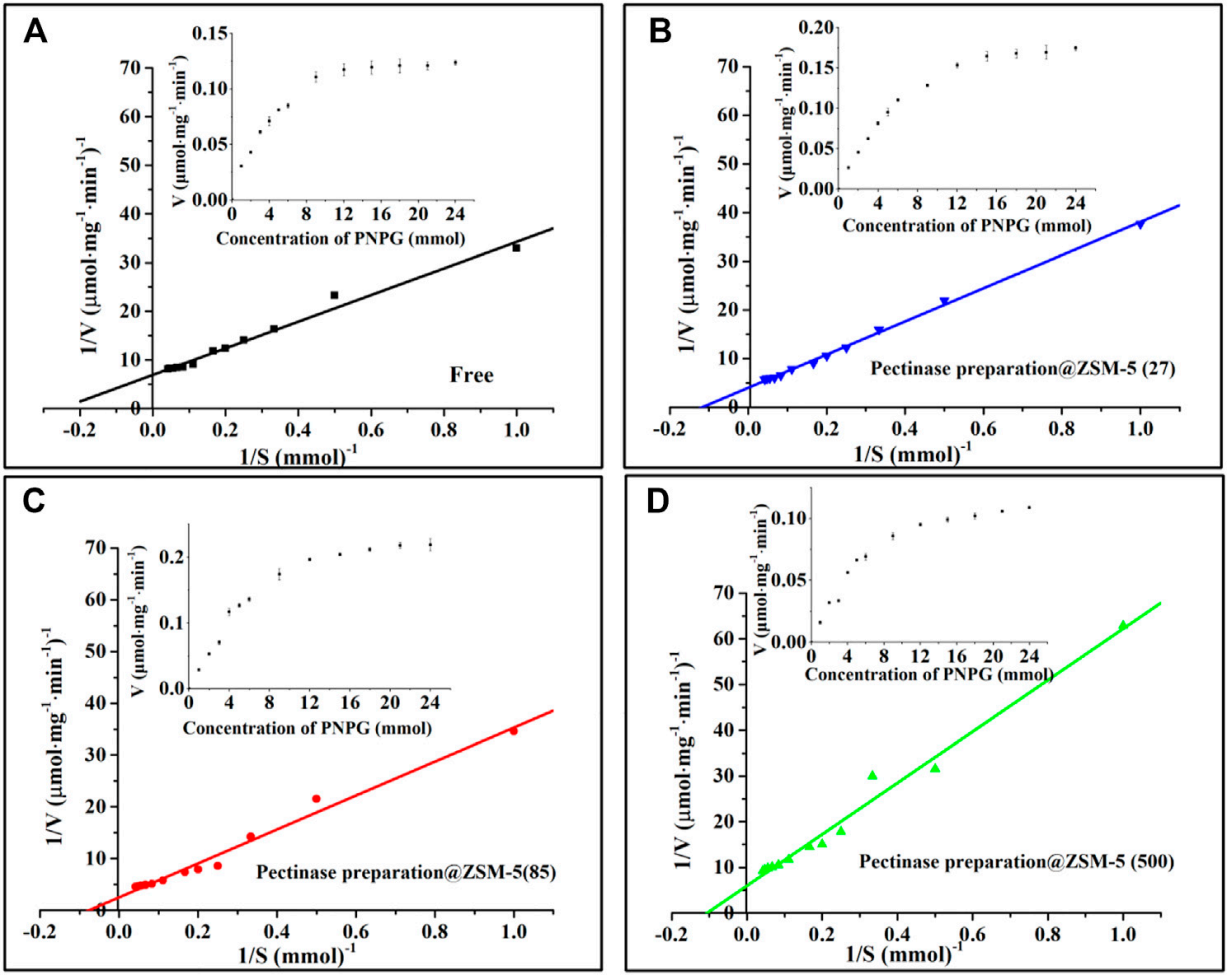
Hanes–Woolf double-reciprocal plots: **(A)** free enzyme, **(B)** pectinase preparation@ZSM-5(27), **(C)** pectinase preparation@ZSM-5(85), and **(D)** pectinase preparation@ZSM-5(500). The inserts are substrate saturation curves for the enzyme-catalyzed reactions.

A plot of the enzymatic reaction rate versus substrate concentration (Figure 4, insert) is a rectangular hyperbolic curve, which is characteristic of Michaelis–Menten enzyme kinetics (English et al., 2006; Reuveni et al., 2014). Based on the Michaelis–Menten equation, we obtained Hanes–Woolf double-reciprocal plots (Figure 4) and calculated the kinetic parameters, namely, the maximal reaction velocity (*V*_max_) and Michaelis–Menten constant (*K*_m_), of the immobilized and free pectinase preparation (Table 1) (Robin et al., 2018); *V*_max_ corresponded to the reaction rate under the condition that the substrate was saturated with pectinase preparation molecules, and *K*_m_ was defined as the substrate concentration that gave a reaction rate equal to half of *V*_max_. We noted that the *K*_m_ values of all the pectinase preparation@ZSM-5 catalysts were higher than that of free pectinase preparation, which indicated that immobilization of pectinase preparation on the ZSM-5 support modulated the kinetic characteristics and the pectinase preparation microenvironment. The *K*_m_ value of the immobilized enzyme was higher than that of the free enzyme, indicating the decreased affinity of the immobilized enzyme to the substrate. The decrease in pectinase preparation–substrate interaction was most likely because of the reduced free movement of pectinase preparation after binding to the carrier or compromised enzyme flexibility that affected the enzyme-substrate binding (Gupta and Raghuvanshi, 2010; Shankar et al., 2015; Singh et al., 2017). Additionally, pectinase preparation@ZSM-5(85) possessed a higher *V*_max_ than the other forms of pectinase preparation@ZSM-5 and free pectinase preparation, and its enzyme activity was 83% higher than that of free pectinase preparation. Immobilized enzymes had higher *V*_max_ values than those of the free enzymes, and an increase in *V*_max_ could be due to the activation of their catalytic active centers because of a change in the enzyme conformation caused by immobilization (Ali Noma et al., 2021), which increased the substrate-to-product conversion rate.

### Ethanol Tolerance of Pectinase Preparation@ZSM-5

**FIGURE 5:**
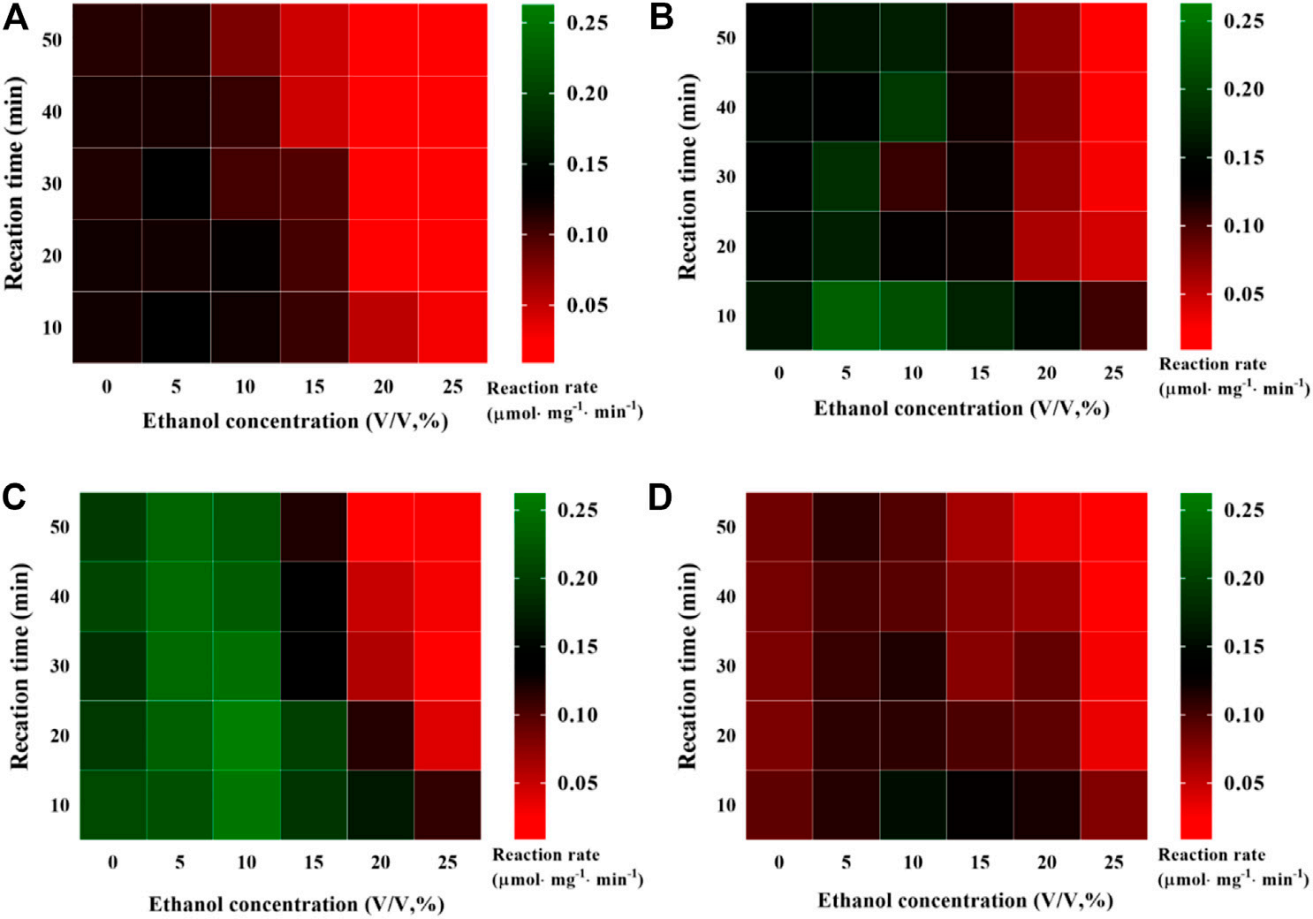
Evaluation of ethanol tolerance. Effect of ethanol concentration on hydrolysis activity of **(A)** free pectinase preparation, **(B)** pectinase preparation@ZSM-5(27), **(C)** pectinase preparation@ZSM-5(85), and **(D)** pectinase preparation@ZSM-5(500).

Good tolerance toward ethanol is critical for the industrial application of enzymes because adding ethanol into the reaction system is helpful for the dissolution of nonpolar substrates (Ogino, 2009; Cheng et al., 2020). Therefore, we investigated the effect of ethanol on enzyme activity, and the results were presented as heatmaps (Figure 5). In the plots, the deeper green indicates higher enzyme activity, whereas the deeper red indicates lower enzyme activity. Interestingly, we observed that the activities of the free pectinase preparation and pectinase preparation@ZSM-5 increased slightly at low ethanol concentrations in the range of 5–10 vol%. For example, the pectinase preparation@ZSM-5(85) activity increased by 19% when the ethanol concentration was 10% (v/v) for a reaction time of 50 min and 50°C as the reaction temperature, compared to that in the absence of ethanol. This was because the ethanol lowered the dielectric constant of the enzyme reaction system and formed hydrogen bonds with water molecules, thus destroying the hydration layer on the surface of the pectinase preparation protein and enhancing the hydrophobic interactions between the nonpolar groups of pectinase preparation and the substrates (Overberger and Meenakshi, 1984; Pazhang et al., 2006; Kumar et al., 2012; Robinson et al., 2014). However, the pectinase preparation activity began to decrease when ethanol concentration exceeded 10% (v/v) and a dramatic decrease in activity was observed at an ethanol concentration of 25%. These negative effects on enzyme activity were observed because high concentrations of ethanol induced denaturation of the pectinase preparation protein (Stepankova et al., 2013). Although a low ethanol concentration during incubation can increase the activity of pectinase preparation, the activity decreased gradually with incubation time (Figure 5). Note that all the pectinase preparation@ZSM-5 catalysts exhibited improved ethanol tolerance compared to free pectinase preparation. Among these, pectinase preparation@ZSM-5(85) showed the best ethanol tolerance, with a 247% higher activity than that of free pectinase preparation under the same conditions (ethanol concentration 10%).

### Engineering Application of Pectinase Preparation@ZSM-5: Baicalin Hydrolysis

**FIGURE 6:**
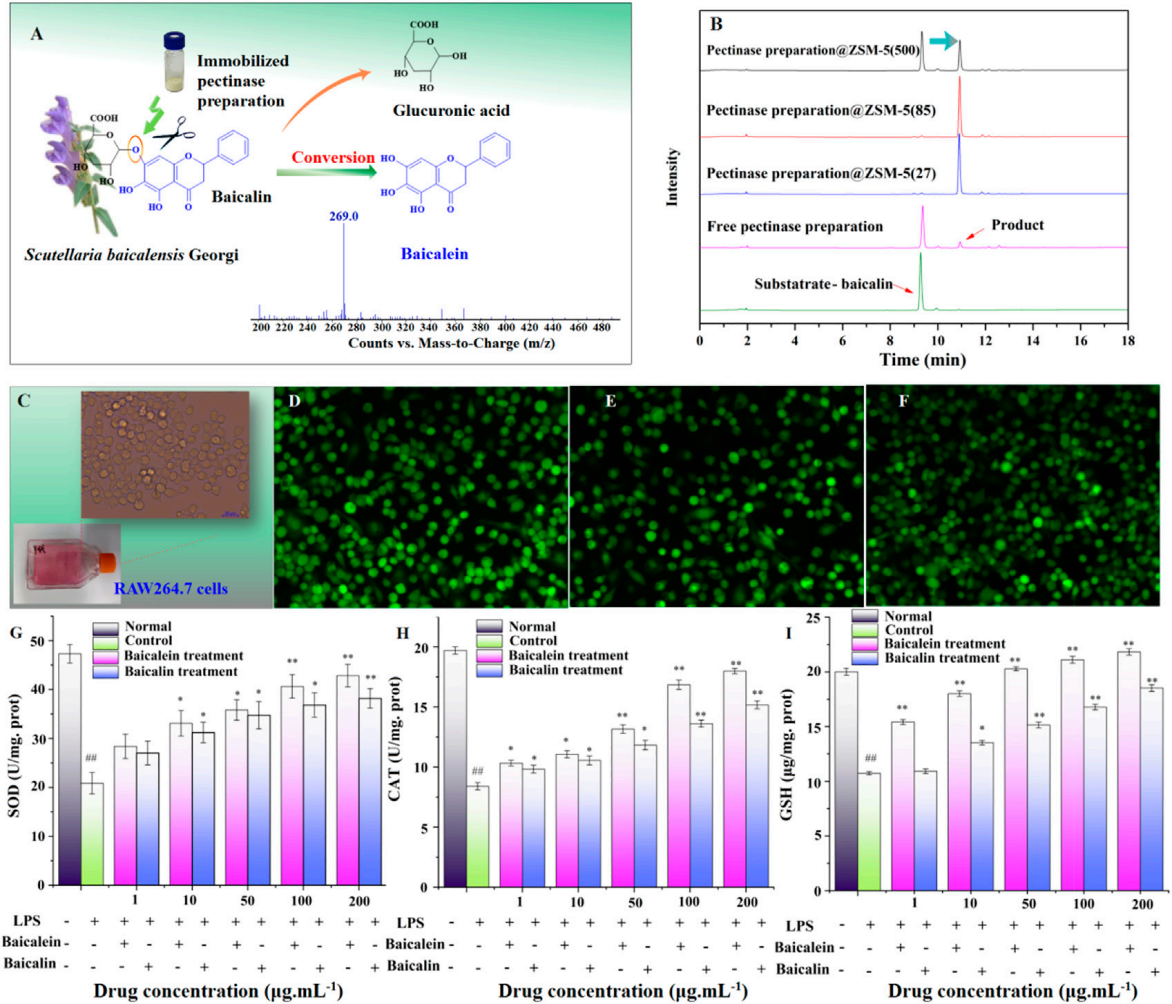
Practical engineering application of pectinase preparation@ZSM-5: enzymatic hydrolysis of baicalin into baicalein. **(A)** Schematic illustration of the hydrolysis of baicalin to form baicalein using pectinase preparation@ZSM-5. **(B)** HPLC spectra of baicalin and as-prepared baicalein. **(C)** Photograph of RAW264.7 murine macrophage cells. **(D–F)** Fluorescence probe confocal microscopy images of **(D)** LPS-injured RAW 264.7 cells, **(E)** baicalin-treated LPS-injured RAW 264.7 cells, and **(F)** baicalein-treated LPS-injured RAW 264.7 cells. **(G–I)** Effect of baicalin and baicalein on **(G)** superoxide dismutase (SOD), **(H)** catalase (CAT), and **(I)** cellular glutathione (GSH) contents of LPS-injured RAW264.7 cells. Significant differences are indicated as follows: ^#^ *p* < 0.05 or ^##^ *p* < 0.01 for the difference between the experimental condition and the 0.9% NaCl control, and *p* < 0.05 or **p < 0.01 for the difference between the experimental condition and the LPS-injured RAW 264.7 control cells.

To evaluate the practical engineering applicability of pectinase preparation@ZSM-5, we used pectinase preparation@ZSM-5 as a biocatalyst to hydrolyze the β-glycosidic bonds in baicalin, a typical flavonoid glycoside extracted from traditional Chinese medicinal herb *Scutellaria baicalensis* Georgi, to produce baicalein, an aglycone flavonoid (Figure 6A).

We used pectinase preparation catalysts to hydrolyze baicalin in 10% ethanol (v/v), which promotes the dissolution of nonpolar baicalin in the aqueous reaction system. The results showed a ∼98% conversion efficiency, 320% higher than that of the free enzyme, for two pectinase preparation@ZSM-5 catalysts (Table 2; Figure 6B).

**TABLE 2:** Conversion and reaction rates for enzymatic hydrolysis of baicalin.

Samples	Free pectinase preparation	Pectinase preparation@ZSM-5(27)	Pectinase preparation@ZSM-5(85)	Pectinase preparation@ZSM-5(500)
Conversion [%]	23.15	97.80	97.46	32.93
V [µmol mg^−1^ min^−1^]	0.10	0.42	0.42	0.14

We observed that the obtained flavonoid aglycone, baicalein, showed higher antioxidant activity than baicalin. For example, the as-prepared baicalein exhibited a 30% higher activity in scavenging 2,2-diphenyl-1-picrylhydrazyl (DPPH) radicals than baicalin *in vitro*, which is even higher than that of the conventionally used vitamins C and E (Supplementary Figure 11). The obtained baicalein, compared with baicalin, also showed higher antioxidant activity in lipopolysaccharide (LPS)-injured RAW 264.7 macrophages (Figures 6G–I), confirmed by confocal microscopy with the dichlorofluorescein (DCFH) fluorescence probe (Figures 6C–F).

## Conclusion

/In this study, we successfully immobilized commercial pectinase preparation over porous ZSM-5 *via* a simple combined strategy. We must pay attention to the fact that many commercial enzyme preparations of unpurified pectinases have enzymatic activities from other enzymes, such as β-glucosidase, cellulases and hemicellulases. Therefore, commercial pectinase preparation can degrade both pectin and β-glycosidic bonds. Some studies have reported that commercial pectinase preparation has better degradation capability for β-glycosidic bonds than pure β-glycosidase. The synergistic effect of multiple enzymes in commercial pectinase preparation may enhance the ability to decompose β-glucosidase. The obtained pectinase preparation@ZSM-5 showed ultra-efficient biocatalytic activity for hydrolyzing the β-glycosidic bonds in model substrate 4-nitrophenyl β-D-glucopyranoside and also for hydrolysis of a substrate that is of importance in a practical engineering application. This activity enhancement was attributed to the immobilization of crosslinked pectinase preparation on ZSM-5, which resulted in higher thermal stability, stronger reaction kinetics, longer cycling life, and better ethanol tolerance for pectinase preparation@ZSM-5 than those of free pectinase preparation. These results demonstrate that pectinase preparation@ZSM-5 can act as an efficient biocatalyst for the hydrolysis of β-glycosidic bonds and has potential practical applications in the food and pharmaceutical industries.

## Data Availability

The raw data supporting the conclusion of this article will be made available by the authors, without undue reservation.
