# Supplementary material for: Immobilized Crosslinked Pectinase Preparation on Porous ZSM-5 Zeolites as Reusable Biocatalysts for Ultra-Efficient Hydrolysis of β-Glycosidic Bonds
Source: Front Chem. 2021 Aug 11;9:677868. doi: 10.3389/fchem.2021.677868 (PMC8385667; doi:10.3389/fchem.2021.677868)
Supplement: Supplementary file 1 [file DataSheet1.docx]

**Supplementary Information**

**Immobilized crosslinked pectinase preparation on porous ZSM-5 zeolites as reusable biocatalysts for ultra-efficient hydrolysis of β-glycosidic bonds**

Can Liu^1†^, Liming Zhang^2†^, Li Tan^1†^, Yueping Liu^1^, Weiqian Tian^2^*, Lanqing Ma^1^*

^1^ Key Laboratory for Northern Urban Agriculture of Ministry of Agriculture and Rural Affairs, Beijing University of Agriculture, Beijing 102206, PR China

Email: lqma@bua.edu.cn

^2^ Department of Fibre and Polymer Technology, KTH Royal Institute of Technology, Teknikringen 56, SE-100 44 Stockholm, Sweden

Email: [weiqian@kth.se](mailto:weiqian@kth.se)

^†^ These authors contributed equally to this work

**C**

**B**

**A**


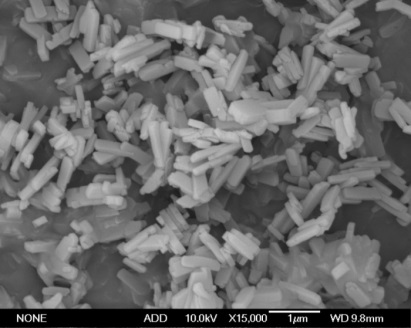

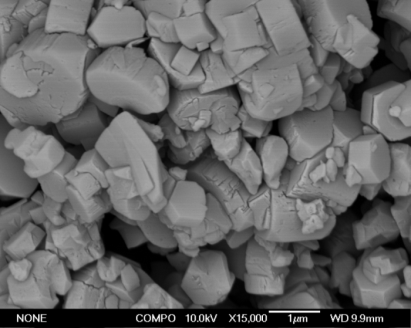

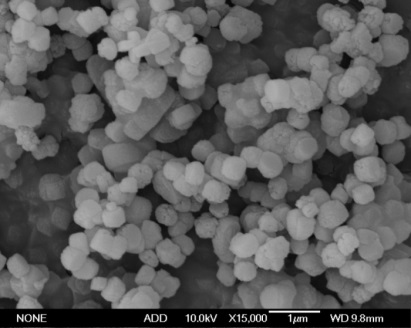


**Figure S1.** SEM images of (A) ZSM-5(27), (B) ZSM-5(85), and (C) ZSM-5(500).

**A**

**B**

**C**

**D**


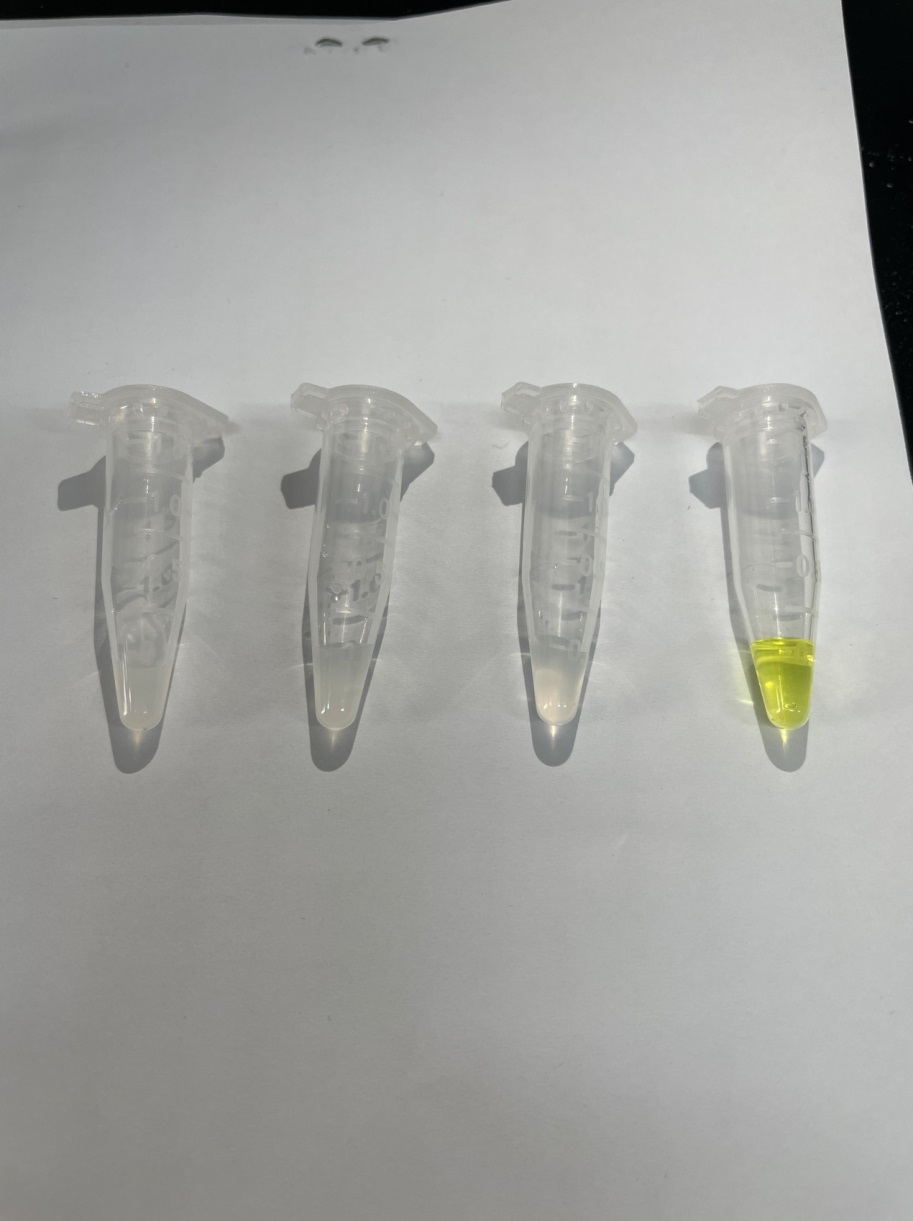


**Figure S2.** PNPG reacted with (A) ZSM-5(27), (B) ZSM-5(85), (C) ZSM-5(500), and (D) free pectinase preparation.

As shown in **Figure S2A–C**, the reaction solution was colorless when PNPG was mixed with the ZSM-5 molecular sieves, which cannot hydrolyze PNPG. However, when PNPG was mixed with pectinase preparation and hydrolyzed by it, the reaction solution became yellow (**Figure S2D**).

**C**

**B**

**A**


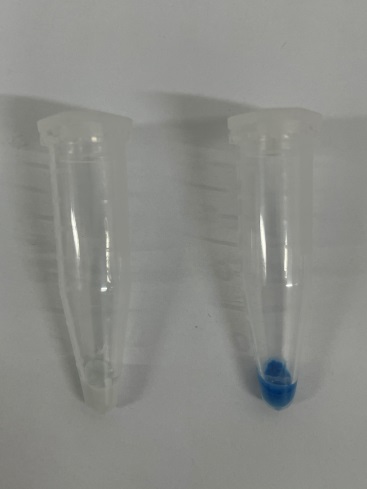

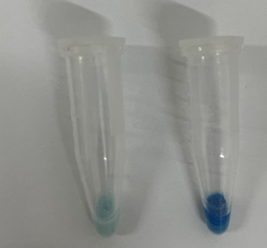

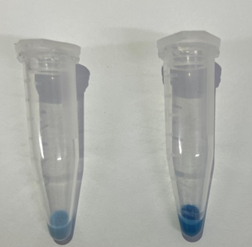


**Figure S3**. ZSM-5 and pectinase preparation@ZSM-5 stained with Coomassie brilliant blue. (A) left: ZSM-5(27), right: pectinase preparation@ ZSM-5(27). (B) left: ZSM-5(85), right: pectinase preparation@ ZSM-5(85). (C) left: ZSM-5(500), right: pectinase preparation@ ZSM-5(500).

As shown in **Figure S3**, when stained with Coomassie brilliant blue, pure ZSM-5(27) was colorless, pure ZSM-5(85) was gray-green, and pure ZSM-5 (500) was bluish grey. All the immobilized enzymes were a dark blue color when they were mixed with Coomassie brilliant blue.





**Figure S4.** Pectinase preparation@ZSM-5 reuse experiments. Pectin was used as the substrate.


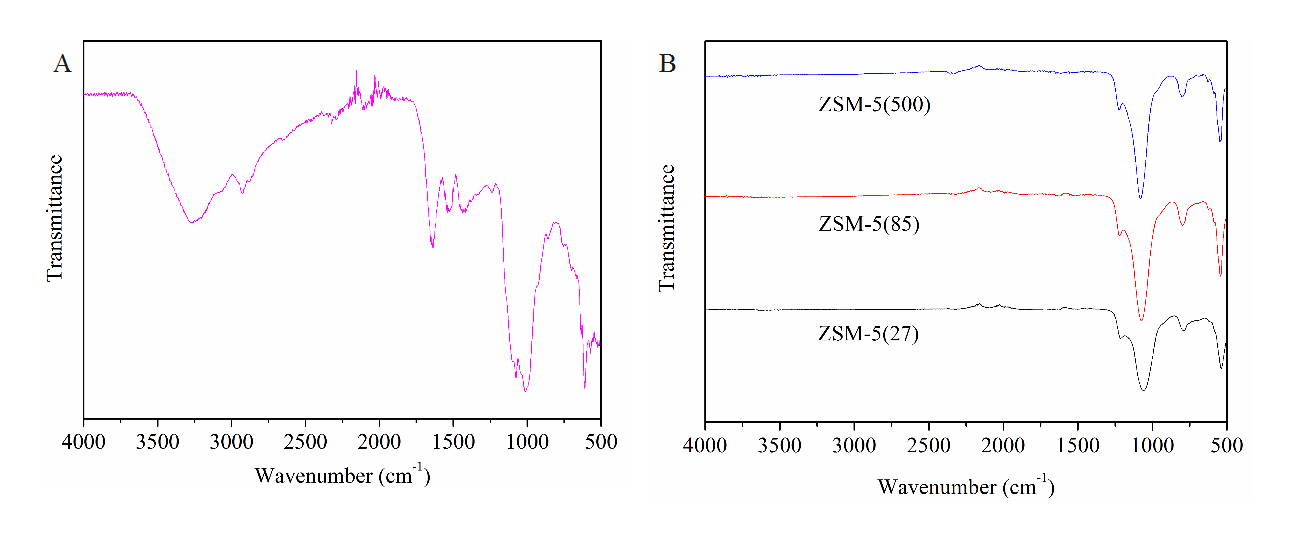


B

**



**

**Figure S5**. FTIR spectra of (A) pectinase preparation and (B) ZSM-5 zeolite supports.





**Figure S6.** XRD patterns of pectinase preparation@ZSM-5(27), pectinase preparation@ZSM-5(85), and pectinase preparation@ZSM-5(500).

The XRD patterns of the immobilized enzymes supported on ZSM-5 structures with Si:Al molar ratios of 27, 85, and 500 are shown in **Figure S6**. The XRD patterns of the three samples are similar. Peaks at 2*θ* values of 7.9, 8.9, 23.1, 23.8, and 24.1 were obtained for each sample. In addition, two adjacent small peaks at 2*θ* values of 44.6° and 45.3°, characteristic peaks of ZSM-5, were also detected. All samples showed MFI structures typical of ZSM-5.


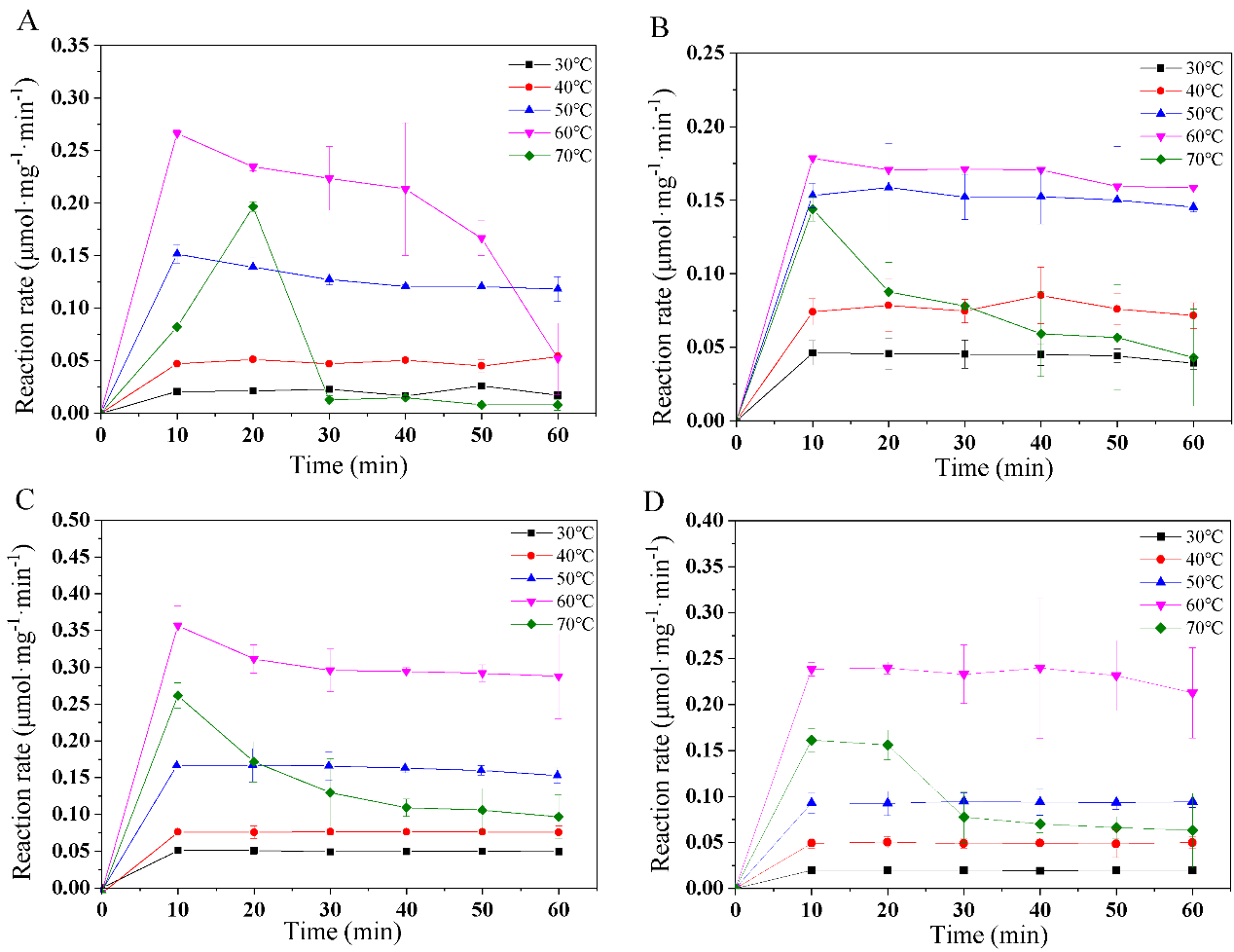


**Figure S7** Effects of reaction temperature and reaction time on enzyme activity (pH=3). (A) free pectinase preparation, (B) pectinase preparation@ZSM-5(27), (C) pectinase preparation@ZSM-5(85), and (D) pectinase preparation@ZSM-5(500).

The enzyme activity was observed to vary with the reaction temperature and reaction time. The free enzyme is unstable, and the enzyme activity is a dynamic process; the free enzyme has the highest activity at 60 °C between 10 and 50 min, but when the reaction time is extended to 60 min, the enzyme activity decreases sharply.

**
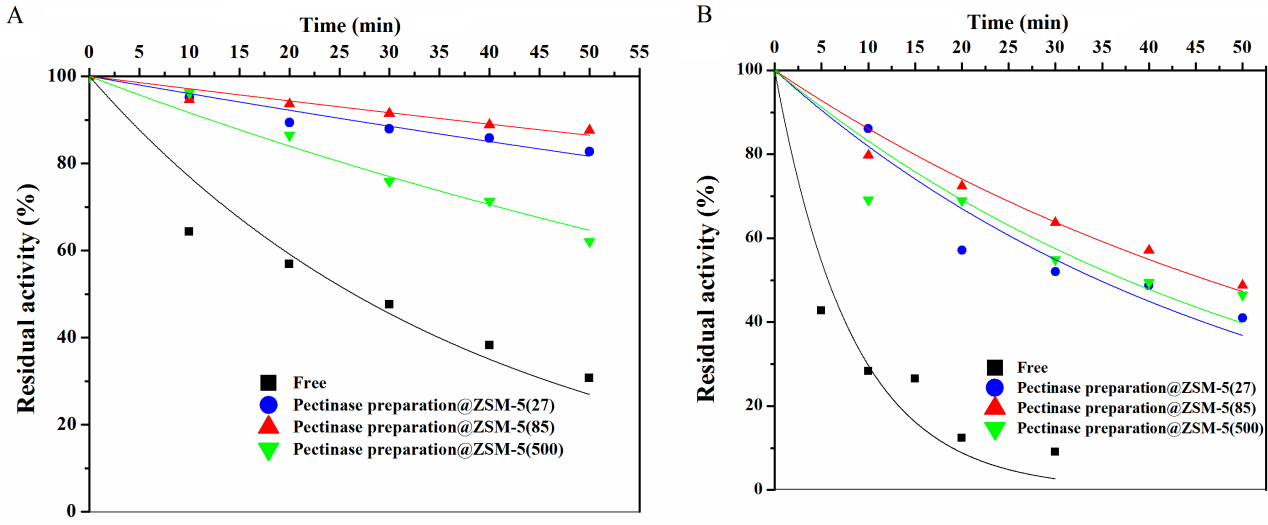
**

**Figure S8.** Residual activity curves of pectinase preparation@ZSM-5 and free pectinase preparation at (A) 60 °C and (B) 65 °C.





**Figure S9. HPLC spectra of baicalin, baicalein, and baicalin reacted with pure ZSM-5.** The three molecular sieve carriers [ZSM-5 (27), ZSM-5 (85), and ZSM-5 (500)] were reacted with the substrate baicalin at 40 °C for 24 hours.

The carrier ZSM-5 cannot catalyze the hydrolysis of baicalin. Each of the three molecular sieve carriers [ZSM-5 (27), ZSM-5 (85), and ZSM-5 (500)] was reacted with the substrate baicalin at 40 °C for 24 h, and we found that the carriers could not hydrolyze the substrate. The above results indicate that the molecular sieves do not have the ability to hydrolyze baicalin; however, the immobilized enzymes pectinase preparation@ZSM-5 [ZSM-5 (27), ZSM-5 (85), and ZSM-5 (500)] can hydrolyze baicalin.

**
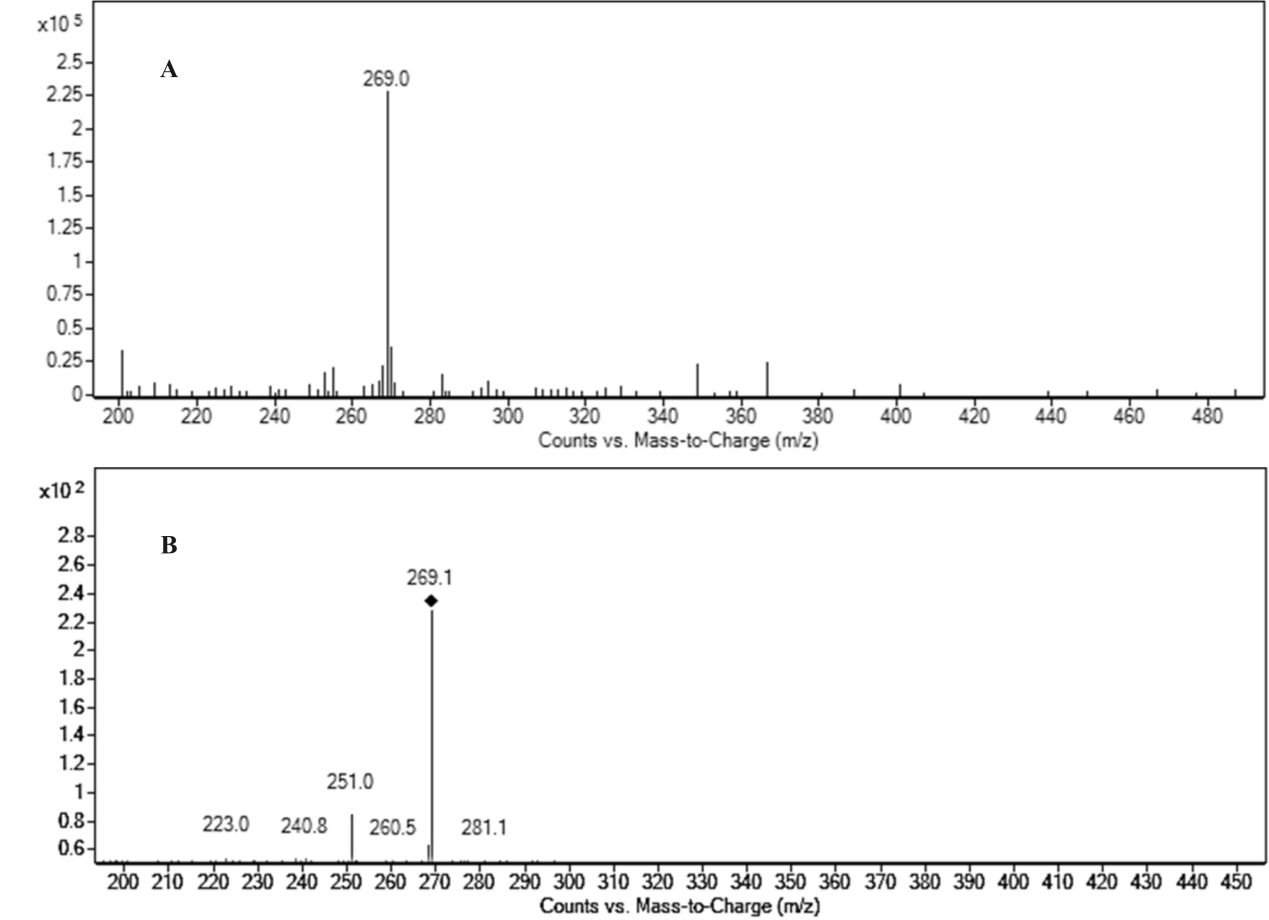
**

**Figure S10.** Mass spectra of as-produced baicalein. (A) The most abundant fragment for baicalein, *m*/*z* 269 [M-H]^-1^, determined via full-scan mode; (B) precursor-to-product ion transitions of *m*/*z* 269 → 251.0 [M-H-H_2_O]^-1^ for baicalein determined by multiple reaction monitoring.

**

**

**Figure S11.** DPPH scavenging capacities of baicalein, baicalin, vitamin C, and vitamin E. The *in vitro* antioxidant activity was evaluated for baicalein and baicalin in terms of their DPPH scavenging capacity. The results indicated that the antioxidant activity of baicalein was higher than those of baicalin, vitamin C, and vitamin E.


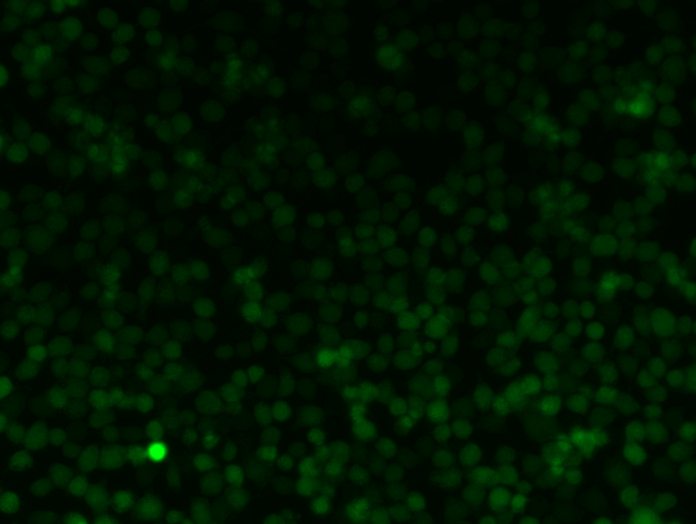


**Figure S12.** Fluorescence microscopy images of untreated RAW264.7 macrophages stained by the DCHF fluorescence probe.

**Table S1** Immobilization yield, immobilization efficiency and recovered activity

| Types of carriers | Total enzyme  (g) | Quality of carrier (g) | Enzyme loading  (g) | U/mg of Biocatalyst | Immobilization efficiency (%) | Recovered activity (%) |
| --- | --- | --- | --- | --- | --- | --- |
| ZSM-5(27) | 3.30 | 1 | 2.04 | 0.17^a^ | 61.80 | 87.57 |
| ZSM-5(85) | 3.30 | 1 | 3.00 | 0.22^a^ | 90.91 | 166.67 |
| ZSM-5(500) | 3.30 | 1 | 2.52 | 0.11^a^ | 76.36 | 70.00 |

^a^ Temperature=50 °C, pH=3

**Table S2** Structural properties of immobilized enzyme particles

| Sample | *S*_BET_^a^  (m^2^·g^-1^) | *S*_ext_^b^ (m^2^·g^-1^) | *S*_micro_^b^  (m^2^·g^-1^) | *V*_tot_^c^  (cm^3^·g^-1^) | *V*_micro_^b^  (cm^3^·g^-1^) | *V*_meso_^d^  (cm^3^·g^-1^) | *D*_p_^e^ (nm) |
| --- | --- | --- | --- | --- | --- | --- | --- |
| Pectinase preparation/ZSM-5(27) | 207 | 28 | 178.32 | 0.096 | 0.068 | 0.028 | 1.861 |
| Pectinase preparation/ZSM-5(85) | 213 | 61 | 151.70 | 0.149 | 0.060 | 0.090 | 2.804 |
| Pectinase preparation/ZSM-5(500) | 317 | 54 | 263.21 | 0.205 | 0.101 | 0.103 | 2.581 |

^a^ *S*_BET_ is the specific surface area calculated using the BET equation.

^b^ *S*_ext_ is the external surface area, *S*_micro_ is the micropore area, and *V*_micro_ is the micropore volume as calculated via *t*-plot analyses.

^c^ Total pore volume *V*_tot_ was determined at 77.3 K and relative pressure *p*/*p*° = 0.9945.

^d^ Mesoporous volume is defined as *V*_meso_ = *V*_tot_ – *V*_micro_.

^e^ Average pore diameter was determined using *D*_p_ = 4*V*_tot_/*S*_BET_.

**Table S3** Acidity of immobilized enzymes as determined by NH_3_-TPD

| **Catalyst** | **Weak acid amount (100–300 °C, mmol·g^-1^)** |  | **Strong acid amount (300–800 °C, mmol·g^-1^)** | **Total acid amount (mmol·g^-1^)** |
| --- | --- | --- | --- | --- |
| Pectinase preparation@ZSM-5(27) | 1.73 |  | 0.97 | 2.71 |
| Pectinase preparation@ZSM-5(85) | 0.41 |  | 2.37 | 2.78 |
| Pectinase preparation@/ZSM-5(500) | 1.59 |  | 0.90 | 2.50 |
